# COVID-19 Vaccine Decision-making Factors in Racial and Ethnic Minority Communities in Los Angeles, California

**DOI:** 10.1001/jamanetworkopen.2021.27582

**Published:** 2021-09-30

**Authors:** Savanna L. Carson, Alejandra Casillas, Yelba Castellon-Lopez, Lisa N. Mansfield, D’Ann Morris, Juan Barron, Ejiro Ntekume, Raphael Landovitz, Stefanie D. Vassar, Keith C. Norris, Steven M. Dubinett, Nanibaa’ A. Garrison, Arleen F. Brown

**Affiliations:** 1 Division of General Internal Medicine and Health Services Research, Department of Medicine, David Geffen School of Medicine, University of California, Los Angeles; 2 Department of Family Medicine, UCLA David Geffen School of Medicine, University of California, Los Angeles; 3 Division of Infectious Disease, David Geffen School of Medicine, University of California, Los Angeles; 4 Olive View-UCLA Medical Center, Sylmar, California; 5 Division of Pulmonary and Critical Care, Department of Medicine, David Geffen School of Medicine, University of California, Los Angeles; 6 Department of Medicine, Veterans Affairs Greater Los Angeles Healthcare System, Los Angeles, California; 7 Jonsson Comprehensive Cancer Center, University of California, Los Angeles; 8 Institute for Society & Genetics, College of Letters and Science, University of California, Los Angeles; 9 Institute for Precision Health, Department of Medicine, David Geffen School of Medicine, University of California, Los Angeles

## Abstract

**Importance:**

The COVID-19 pandemic has had disproportionate effects on racial and ethnic minority communities, where preexisting clinical and social conditions amplify health and social disparities. Many of these communities report lower vaccine confidence and lower receipt of the COVID-19 vaccine. Understanding factors that influence the multifaceted decision-making process for vaccine uptake is critical for narrowing COVID-19–related disparities.

**Objective:**

To examine factors that members of multiethnic communities at high risk for COVID-19 infection and morbidity report as contributing to vaccine decision-making.

**Design, Setting, and Participants:**

This qualitative study used community-engaged methods to conduct virtual focus groups from November 16, 2020, to January 28, 2021, with Los Angeles County residents. Potential participants were recruited through email, video, and telephone outreach to community partner networks. Focus groups were stratified by self-identified race and ethnicity as well as age. Transcripts were analyzed using reflexive thematic analysis.

**Main Outcomes and Measures:**

Themes were categorized by contextual, individual, and vaccine-specific influences using the World Health Organization’s Vaccine Hesitancy Matrix categories.

**Results:**

A total of 13 focus groups were conducted with 70 participants (50 [71.4%] female) who self-identified as American Indian (n = 17 [24.3%]), Black/African American (n = 17 [24.3%]), Filipino/Filipina (n = 11 [15.7%]), Latino/Latina (n = 15 [21.4%]), or Pacific Islander (n = 10 [14.3%]). A total of 39 participants (55.7%) were residents from high-poverty zip codes, and 34 (48.6%) were essential workers. The resulting themes included policy implications for equitable vaccine distribution: contextual influences (unclear and unreliable information, concern for inequitable access or differential treatment, references to mistrust from unethical research studies, accessibility and accommodation barriers, eligibility uncertainty, and fears of politicization or pharmaceutical industry influence); social and group influences (inadequate exposure to trusted messengers or information, altruistic motivations, medical mistrust, and desire for autonomy); and vaccination-specific influences (need for vaccine evidence by subpopulation, misconceptions on vaccine development, allocation ambiguity, vaccination safety preferences, the importance of perceiving vaccine equity, burden of vaccine scheduling, cost uncertainty, and desire for practitioner recommendation).

**Conclusions and Relevance:**

In this qualitative study, participants reported a number of factors that affected their vaccine decision-making, including concern for inequitable vaccine access. Participants endorsed policy recommendations and strategies to promote vaccine confidence. These results suggest that support of informed deliberation and attainment of vaccine equity will require multifaceted, multilevel policy approaches that improve COVID-19 vaccine knowledge, enhance trust, and address the complex interplay of sociocultural and structural barriers to vaccination.

## Key Points

**Question:**

What factors do members of multiethnic communities at high risk for COVID-19 infection and morbidity in Los Angeles County, California, cite as influencing vaccine decision-making and acceptability?

**Findings:**

In this qualitative study, 70 participants from racial and ethnic minority communities in Los Angeles County described a complex vaccination decision-making process influenced by misinformation and politicization, deep apprehension related to historical inequity and mistreatment, access barriers related to social disadvantage, and a need for community engagement and trusted messengers.

**Meaning:**

This study suggests that COVID-19 vaccine equity will require multifaceted policies and programming that respect community concerns and the need for informed deliberation, invest in community-based engagement, improve accessibility and transparency of information, and reduce structural barriers in vaccination.

## Teaser

This qualitative study conducted virtual focus groups to explore barriers and facilitators to COVID-19 vaccine readiness reported by members of disproportionately affected multiethnic communities in Los Angeles County, California.

## Introduction

Increasing COVID-19 vaccine uptake is essential to reducing COVID-19 disparities, but it requires understanding the process and needs within vaccine decision-making. Vaccine decision-making, including deliberation, describes weighing the pros and cons of vaccine efficacy and safety and is a normal, appropriate response to any new treatment or intervention.^1^ In the US, racial and ethnic minority communities have the highest risk of COVID-19 morbidity and mortality yet report lower vaccine confidence and lower receipt of the COVID-19 vaccine.^2,3,4,5^ Medical mistrust, rooted in historical and contemporary racism, has lowered vaccine confidence in racial and ethnic minority groups.^6,7,8,9,10^ However, vaccine uptake is influenced by more than mistrust, including socioeconomic and health inequity.^1,8,9,11^

Because vaccine acceptability is strongly associated with vaccine uptake, understanding factors that influence the multifaceted vaccine decision-making process is critical to narrowing COVID-19–related disparities in racial and ethnic minority communities. It is essential to consider the factors that influence COVID-19 vaccine acceptability and how the decision-making process may differ from other vaccines. These factors link to unique perceptions of disease risk and context, such as one’s emotional state and sociopolitical and environmental influences.^12,13^ Therefore, aspects unique to the COVID-19 pandemic, including the ongoing sociopolitical contexts, mistrust, economic instability, and mental and health challenges, add to the complexity of the vaccine decision-making process.^14^ Given the multilevel nature of vaccine concerns, an inquiry into the commonalities between marginalized groups is required to tailor community engagement approaches.^14,15,16,17,18^

This study explores barriers to and facilitators of COVID-19 vaccine readiness reported by members of disproportionately affected multiethnic communities in Los Angeles County (LAC), California. Exploration of factors in the decision-making process for COVID-19 vaccines can inform public health and policy initiatives for equitable vaccine distribution.

## Methods

### Study Design, Participants, and Setting

We used community-engaged qualitative methods to better understand factors that contribute to vaccine deliberation and acceptability in racial and ethnic minority groups at high risk for COVID-19 infection and morbidity in LAC. Los Angeles County is a uniquely fitting setting to address these questions—it is the most populous and 1 of the most diverse counties, has among the highest number of COVID-19 cases and deaths, and COVID-19–related morbidity and mortality disproportionately affect racial and ethnic minority individuals and high-poverty communities.^19^ The UCLA Institutional Review Board approved the study, and we obtained verbal informed consent from all participants. Although focus group facilitators, moderators, and participants used their personal names within the virtual focus groups, all personal identifiers (names and workplaces) were removed from transcripts by the professional transcription company before analysis. We report our findings using the Standards for Reporting Qualitative Research (SRQR) reporting guideline.^20,21^ A critical and radical paradigm^22^ and community-partnered research methods guided this study. Local community partners and a community advisory board collaborated on study design, recruitment, interpretation, and dissemination.

Between November 16, 2020, and January 28, 2021, we conducted 2-hour virtual focus groups with LAC residents using Zoom.^23^ We recruited individuals virtually through telephone, video conferencing, and email outreach to community partners and networks from communities that face a high risk of COVID-19 morbidity and mortality attributable to race and ethnicity, age, essential worker status, and residential area (median household income <$40 000, 2010 US Census).^19,24,25^ Eligible participants self-identified as American Indian, Black/African American, Filipino/Filipina, Latino/Latina, or Pacific Islander. Focus groups were stratified by race and ethnicity as well as age (≥50 years, <50 years, or mixed age). Of 144 candidates screened, 6 were ineligible because of self-reported White race and ethnicity, and 13 were excluded because of insufficient numbers for a specific racial and ethnic focus group (ie, <3). We ordered and sequentially recruited from the remaining 125 eligible participants by race and ethnicity as well as age, prioritizing essential workers and residents of low-income zip codes. Of 81 invited to participate, 70 participated in 13 focus groups (4-6 per group).

### Data Collection

A semistructured focus group guide was developed from previous qualitative vaccine acceptability studies^26,27,28,29^ with input from community organizations. Question domains on COVID-19 vaccines included concerns, risks, benefits, information sources, trusted entities, barriers, and recommendations for improving access (Box). Participants were asked to contribute as both individuals and experts representing their families and communities.

Box. **Focus Group Question Guide**

- Icebreaker: Please state your name, tribal affiliation (if applicable), current feelings on the pandemic, and 1 word to describe your racial/ethnic community.
- What have you or members from your community heard about any vaccines to protect against COVID-19?
- What concerns do you, your family, or your community have about receiving the COVID-19 vaccine? What additional information do you need to feel comfortable to receive the COVID-19 vaccine?
- When a COVID-19 vaccine is available, who and where would you feel most comfortable getting the vaccine?
- What do you think are some risks and benefits of the COVID-19 vaccine?
- Situational question: It could be the case that some of the vaccines offered may not 100% protect against COVID-19 infection. The vaccine may lower the chances of being infected by COVID-19. Or, if you do get COVID, the vaccine may lower your chances of getting very sick from it (reduce the severity of the disease or reduce additional health complications). However, it may not be perfect, and it may not prevent 100% of people from COVID-19. How would you feel about the information (that getting the vaccine does not 100% protect against being infected)?
- What challenges do you, your family, or people you know may face in getting the COVID-19 vaccine?
- What are some ways to get the COVID-19 vaccine to the people who need it most when it becomes available?

We provided partner organizations with a study description to assist with recruitment. We also described how research outcomes might benefit their respective organizational missions, including strategic planning about COVID-19 education.

To enhance congruency between researchers and participants, in all but 1 focus group, either the moderator, trained as the facilitator, or a community partner, self-identified with the group participants’ race or ethnicity. Each group began by restating the study’s purpose and emphasizing the importance of community voices for understanding COVID-19 vaccine acceptability to shape public health policies. We facilitated an icebreaker where moderators and participants described their community and feelings on the pandemic. Two focus groups were conducted in Spanish, and the other 11 were conducted in English. Several research team members attended each focus group to ensure consistent use of the focus group guide and prompts, facilitate debriefing, and record field notes. Recordings were deidentified, translated, and transcribed by a professional transcription service. Each participant received a $100 gift card and completed an online demographic and attitudes survey.

### Data Analysis

We analyzed transcripts using a critical realist,^30^ reflexive, 6-phase thematic analysis approach^31,32^ in Atlas.Ti (ATLAS.ti Scientific Software Development GmbH). Two experienced coders (S.L.C. and L.N.M.) reviewed the transcripts and field notes to develop a preliminary codebook, then tested and amended the codebook’s initial practicality after the coding of 2 transcripts. The coders reached iterative consensus on the evolving codebook, code definitions, and coding approach and used memos to document thematic evolution throughout the analysis. Triangulation was achieved by reviewing the field notes, holding iterative discussions with all moderators and facilitators, and sharing preliminary results at community partnered meetings to validate perspectives.

We used the Vaccine Hesitancy Matrix (VHM), developed by the World Health Organization Strategic Advisory Group of Experts on Immunization Vaccine Hesitancy Working Group,^33,34^ to categorize prominent themes shared commonly across all racial and ethnic groups and most focus groups. The VHM categorizes vaccine hesitancy determinants into contextual influences (historical, sociocultural, environmental, health system or institutional, and economic or political factors), individual and group influences (personal, social, and peer environment), and vaccine- or vaccination-specific issues (vaccine or vaccination factors).^13,34,35^ Although various vaccine hesitancy definitions, determinants, measures, models, and terms exist,^36,37,38,39^ including arguments for inclusion or exclusion of vaccine social determinants,^40^ the VHM provides a multifaceted picture of vaccine acceptability. The VHM accounts for factors other than hesitancy and vaccine refusal, such as vaccination program design, allowing for systems-level^41^ perspectives in COVID-19 vaccine decision-making.

## Results

A total of 13 focus groups were conducted with 70 participants (50 [71.4%] female). We conducted 3 American Indian focus groups (n = 17), 3 Black/African American groups (n = 17 participants), 2 Filipino/Filipina groups (n = 11), 3 Latino/Latina groups (n = 15), and two Pacific Islander groups (n = 10). A total of 39 participants (55.7%) were residents from high-poverty zip codes, and 34 (48.6%) were essential workers. A total of 31 (44.3%) were employed full time, and 13 (10.0%) were unemployed and 8 (11.4%) retired. A total of 37 (52.9%) reported they were likely or very likely to receive the vaccine when available. Demographic characteristics and survey responses are given in Table 1 and eTables 1 and 2 in the Supplement.

**Table 1.** Focus Group Participant Demographic Characteristics (N = 70)

Characteristics	Finding^a^
Race and ethnicity, by focus group participation	
American Indian	17 (24.3)
Black/African American	17 (24.3)
Filipino/Filipina	11 (15.7)
Latino/Latina	15 (21.4)
Pacific Islander	10 (14.3)
Age, y	
<50	38 (54.3)
≥50	32 (45.7)
Sex	
Female	50 (71.4)
Male	19 (27.1)
Other	1 (1.4)
Educational level	
Some high school	5 (7.1)
High school graduate or GED	5 (7.1)
Associate or technical degree	17 (24.3)
Bachelor degree	19 (27.1)
Graduate degree	22 (31.4)
Prefer not to answer	2 (2.9)
No. of people in household, mean (SD)	3.04 (2.2)
Employment status	
Full-time	31 (44.3)
Part-time	18 (25.7)
Unemployed	13 (10.0)
Retired	8 (11.4)
Essential worker	34 (48.6)
Resides within a low-income zip code (median annual household income <$40 000, per US Census 2010)	39 (55.7)
Very important or important for all people in community to receive the COVID-19 vaccine	54 (77.1)
Very likely or moderately likely to get an approved COVID-19 vaccine when available	37 (52.9)

Abbreviation: GED, general educational development.

^a^ Results are No. (%) unless otherwise stated.

Participants described influences in their vaccine decision-making process. We organized results using the VHM constructs, reporting themes, subthemes, and quotes in Table 2. The specificity and comprehensiveness of the VHM worked well to frame results by accounting for interrelated socioeconomic and contextual factors occurring during the COVID-19 pandemic. Notably, most of the resulting themes portray dimensions within potential vaccine inequity for each VHM category.

**Table 2.** Themes, Subthemes, and Salient Quotes for COVID-19 Vaccine Acceptability and Deliberation Within Racial and Ethnic Minority Communities Using World Health Organization Vaccine Hesitancy Matrix Categories

Theme	Quotes
**Contextual influences (influences arising from historical, sociocultural, environmental, health system or institutional, and economic or political factors)**	
Unclear and unreliable information	“The Hispanic community needs information … not from social media but truthful information, from newspapers or information channels that provide authentic or more reliable information.”—Latino/Latina participant (focus group 12)
	“If you could get healthcare providers, even if it’s nurses or nurse practitioners or something, that’s Native that comes out and talks to the communities…, it would give them peace.”**—**American Indian participant (focus group 10)
References to mistrust from historical or contemporary unethical research studies	“There’s certainly sort of that general feeling among indigenous peoples that a lot of times research hasn’t been with the benefit of those people in mind. In fact, almost the opposite.”**—**Pacific Islander participant (focus group 7)
	“I don’t want to call it a ‘dog whistle,’ but just to hear that somehow what’s being discussed as, you know, the priorities of the African American communities as if the African American communities aren’t aware of the past experiments, whether it’s social science, medical that we have been a part of unknowing what the truth was behind it and the long-lasting effects that it’s had on our families, our men, women and children… We don’t want to be another Tuskegee Experiment or something else. We love that you’re thinking about us, but you know.”**—**Black/African American participant (focus group 6)
Concern for inequitable access or differential treatment	“We know when something is rolled out much nicer, much more organized services of a better quality, [and] when people are treated differently in other areas… if I find that what’s happening in South Central Watts… I’m going to have a problem.”**—**Black/African American participant (focus group 6)
	“We hope we get same vaccine that the healthcare staff are getting, we hope it will be the same for us.”**—**Latino/Latina participant (focus group 8)
	“Trump and his band of merry men have gotten some magical serum that had him jump out of the bed. Why can’t we all have that? … Why is it only for the elite? ... Why can’t we all have the same vaccination? What’s in this needle that wasn’t in his?”**—**Black/African American participant (focus group 5)
	“Access… there are so many issues with it, what if they’re pushed to the back of the line?”**—**American Indian participant (focus group 3)
	“The gatekeepers, typically are the people at the front desk… dealing with them is hard … sometimes the hospitality isn’t there and that’s a big barrier.”**—**Black/African American participant (focus group 6)
Accessibility barriers	“In terms of translators… maybe those who may not be of the same culture or the way that they’re explaining the medical terminology may be intimidating.”**—**Filipino/Filipina participant (focus group 9)
	“If they do qualify for the vaccine they’d have to take a day off of work to go. And not everybody has the privilege of sick hours or anything like that. So, they would sacrifice a day’s wage to go get the vaccine.”**—**Latino/Latina participant (focus group 13)
Accommodation barriers	“Some elders… they’re houseless… there’s no cell phone, they ride public transportation. How is it going to get distributed [to them]? I would think it would go to the most vulnerable… [but] it feels like our culture always gets the shitty end of the stick.”**—**American Indian participant (focus group 4)
	“Is the federal and local government prepared to support the people in the event that the side effects of taking this vaccine prevents them from being able to go to work… people are begging for another stimulus check just to make sure that they have food ... so, what does it look like if people are affected to the extent to where they cannot continue to even work?”**—**Black/African American participant (focus group 6)
Eligibility uncertainty	“In my community there’s a good chunk of people without insurance, and also that serves as a barrier. I don’t know how much the vaccine is without insurance ... Filipinos can get lost in the system because, like I said before, the language barriers and me, personally, in my family, just not knowing how things work in the system.”**—**Filipino/Filipina participant (focus group 9)
Fears of politicization or pharmaceutical industry influence	“Watching the political race unfold and hearing the different sound bites from different candidates regarding the speed [of the vaccine] and how it would be done on time. So that placed a lot of doubt in people, because we heard if it’s done in a certain window then it’s too fast and just different… political agendas.”**—**American Indian participant (focus group 4)
**Individual and group influences (influences arising from the personal perception of the vaccine or social or peer environment)**	
Inadequate exposure to trusted messengers or information	“It’s difficult to build trust in the community when people hear things that are not true … but we build that trust by being truthful about what we do and say on the streets. It’ll be… setting up tables to give out information about vaccines, making appointments, where and when they can go, and have flyers saying ‘If you want, we can make the appointment for you,’ and have appointment letters and do all that. Whoever goes [to the communities] or gives information in support of the health department will be great.”**—**Latino/Latina participant (focus group 11)
Altruistic motivations	“Optimism will lead people to get vaccinated, to take care of themselves, to keep going ... we should talk about life to a certain extent because right now the information is chaotic and I’ve found something positive for me, for my family and also for my community.”**—**Latino/Latina participant (focus group 11)
Medical mistrust	“That had to do with institutional mistrust… if there are options to get the vaccine that are a little less institutional might go a long way to building trust with the communities who would maybe need to be a little bit more persuaded.”**—**Filipino/Filipina participant (focus group 1)
Desire for autonomy	“I hope that people are sensitive to the fact that communities have mistrust of medicine and not to treat them as being, oh, you’re just being silly… just take this. I mean, for people who have been let down by the system in the past, I would hope that there is a little bit of compassion and understanding and patience, and not treating someone as less than because they are impacted by situations in the past.”**—**American Indian participant (focus group 3)
**Vaccine or vaccination—specific issues (directly related to vaccine or vaccination)**	
Need for vaccine evidence by subpopulation	“The other concern is the long-term effects and not sampling enough Pacific Islanders, women, people of color, those with health disparities.”**—**Pacific Islander participant (focus group 2)
	“There has been a lot of concerns in my family on how the vaccine works for people with heart disease, which really affects also a lot of the Filipino community, and also those with respiratory diseases.”**—**Filipino/Filipina participant (focus group 9)
	“Most of these trials have been done on predominantly—at least from my belief, from what I’m seeing and hearing is that—it’s being predominantly done on White/Caucasian people. And although I look that way, how do I know that that’s not going to affect me or affect my people in a different way and my family?”**—**Filipino/Filipina participant (focus group 1)
Vaccine development misconception	“Research and data… are very intimidating topics… if you want to build rapport and build those connections, maybe inviting Pacific Islanders to have a seat at the table in the very beginning of the [vaccine] development process.”**—**Pacific Islander participant (focus group 2)
	“Back to the point of the testing feeling like it’s being rushed, I feel like right now whoever tries it is literally going to be the test group… like the same thing with Apple launches. I never want to get the first phone. I want to get the one that after all the bugs get worked out. I’ll go get that one.”**—**Filipino/a participant (focus group 1)
Allocation ambiguity	“You have families who have no electronic devices ... putting this [vaccine registration] now on them with the overhead of ‘COVID is going to come get you if you don’t do this,’ it’s going to be quite traumatic for a lot of our communities.”**—**American Indian participant (focus group 10)
Cost uncertainty	“It is very disheartening… we know the rich [will] get it because they have the money.”**—**Filipino/Filipina participant (focus group 1)
Vaccination safety preferences	“Undocumented people are afraid to go to the doctor as if they were forbidden to do that for fear that they are a public burden. ‘You can’t. You don’t have…’ So, is the vaccine going to be free as the COVID test right now?”**—**Latino/Latina participant (focus group 8)
Importance of perceiving equity across one’s community	“Our incarcerated community members ... elders… the foster care system. We can’t forget about them. What are those agencies going to do ... to make sure that they’re taken care of, as well?”**—**American Indian (focus group 4)
	“Homeless. That’s another group that should be taken into account so that they can also be protected. We protect ourselves, but they don’t have any protection. They are, like people say … sailing against the wind. There’s no fairness… they should be counted.”**—**Latino/Latina participant (focus group 8)
Burden of vaccine schedule on caregivers and families	“Resources are limited… if only a third of the family is eligible right now because of the phase that they’re in to get the vaccine, will they go and get it, or will they wait till the entire family can go and get it, so it’s all just one swoop?”**—**American Indian participant (focus group 10)
Desire for practitioner recommendation	“In regards to how the vaccine affects persons with different underlying health issues when I read it says ‘Talk to your doctor’ ... but we all have different types of doctors and different types of insurances, depending on how good your insurance may or may not be depends on how maybe proactive your doctor may be in providing information. So I think that’s a concern … how it affects underlining conditions, whether it’s thyroid, diabetes, et cetera ... I haven’t seen any massive information related to that.”**—**Black/African American participant (focus group 12)

### Contextual Influences

#### Unclear and Unreliable Information

Participants described conflicting vaccine information in the news, social media, and from leaders, likely stemming from an absence of factual information, misinformation, and a scarcity of trusted messengers or sources. A Latino/Latina participant (focus group 12) explained, “These kinds of decisions should be informed decisions after getting information from authentic sources of information.”

#### References to Unethical Historical or Contemporary Research Leading to Mistrust

Specific examples of unethical historical or contemporary research affecting one’s community negatively influenced trust in COVID-19 vaccine research. Consequentially, many expressed hesitation about being among the first to be vaccinated, and several expressed fears of experimentation. A Black/African American participant (focus group 5) described a “general unease,” which others endorsed because of previous unethical research, mistreatment, experimentation, or discrimination. Participants requested acknowledgment, empathy, and understanding of current and historical events that led to their communities’ mistrust.

#### Concern for Inequitable Access or Differential Treatment in Vaccination

All groups expressed deep concerns about potential inequity in vaccine management, distribution, access, and quality. Participants feared receiving differential treatment, projecting that well-resourced communities, White people, and the “rich and powerful” (Filipino/Filipina participant from focus group 1) would be the first to receive a vaccine and would receive higher-quality or better treatment during vaccination.

#### Accessibility Barriers, Accommodation Barriers, and Eligibility Uncertainty

Several social determinants of health were identified as barriers to vaccine access (availability and quality of translation services as well as limited technology or internet access to register), vaccination accommodations (limited transportation, a lack of employment benefits, including paid time off for vaccination, or adverse effects), or vaccine eligibility (uninsured or undocumented). An American Indian participant (focus group 10) described a “logistical nightmare” to access transportation for vaccination.

#### Fear of Political or Pharmaceutical Industry Influences

Participants described political influences in vaccine development and pharmaceutical companies’ interests, motives, and profits. A Filipino/Filipina participant (focus group 9) described limited “trust in politics” as a barrier to vaccination.

### Individual and Group Influences

#### Inadequate Exposure to Trusted Messengers or Information

Many felt compelled to consider receiving the vaccine to protect themselves or others but expressed uncertainty because of insufficient information, including a lack of opportunities to discuss vaccine concerns. Facing this dilemma, many wanted to wait. Participants desired notification and communication about the COVID-19 vaccine from their medical practitioners, local health centers, and community leaders. A Pacific Islander participant (focus group 2) explained, “There’s definitely some key leaders in each of our communities. Some of them may be faith leaders.”

#### Altruistic Motivations

Participants desired outreach strategies that promoted altruistic vaccination motivations, including how the vaccine may “protect us all” from infection to themselves or communities (Latino/Latina participant in focus group 8). Participants were optimistic about the potential of reducing stress as well as infection risks and desiring a return to social and cultural norms.

#### Medical Mistrust

Some participants described medical mistrust, worried about overmedicalization, or referenced past medical experiences, including medical errors, mistreatment, and racism in health care. As such, participants requested sensitive, respectful, and equitable treatment during vaccination.

#### Desire for Autonomy

Some worried that vaccines would become mandatory and expressed a desire for autonomy “to be respected” around informed decision-making, particularly given the current uncertainties regarding themselves or their community’s safety (American Indian participant in focus group 4).

### Vaccine and Vaccination-Specific Influences

#### Need for Vaccine Evidence by Subpopulation

Many expressed a belief that the vaccine clinical trials primarily included healthy, young, and White participants. They wanted to see clinical trial demographic characteristics and evidence of the vaccine’s effectiveness and safety outcomes, requesting data representing the same racial and ethnic community, age group, and health conditions. A Black/African American participant (focus group 5) stated interest in “any data that’s connected with some of the conditions that are prevalent in our community.”

#### Vaccine Development Misconception

Because of the development of multiple vaccines, participants questioned whether they should wait for future iterations with improved safety and efficacy profiles. Participants questioned the rapid vaccine development process and whether the scientific or testing process had been rushed. A Black/African American participant (focus group 12) asked, “Is this like the rough draft?”

#### Allocation Ambiguity and Cost Uncertainty

The allocation process seemed unclear, including notification and requirements, insurance coverage, or out-of-pocket costs. A Pacific Islander participant (focus group 2) stated that uncertainties are “tied to not having enough information.”

#### Vaccination Safety Preferences

Preferences for vaccination location included familiar or local sites instead of a mass vaccination site with long lines or crowds that would increase COVID-19 exposure risk. Additional site concerns included a lack of accommodations for disabled, elderly, or immobile people. Others worried large sites would lack proper medical attention to monitor adverse effects with too much “room for error” (Latino/Latina participant in focus group 13). Although local, familiar sites were preferred, participants worried about vaccination infrastructure within underresourced communities (ie, a trusted site may lack suitable freezers for vaccine storage).

#### Importance of Perceiving Equity Across One’s Community

Participants emphasized the importance of equitable vaccine allocation in their communities but feared certain groups would be left behind, including “low-income, homeless,” bedridden, or incarcerated individuals (Latino/Latina participant in focus group 13).

#### Burden of Vaccine Schedule on Caregivers or Families

Dual-dose vaccine schedules were seen as an additional burden for families or caregivers, those “having to take all the kids,” those with limited transportation access, or those with other mobility obstacles (American Indian participant in focus group 4).

#### Desire for Health Care Practitioner Recommendation

Participants with underlying health conditions or concerns expressed a desire for a practitioner recommendation for vaccination. A Pacific Islander participant (focus group 7) explained, “I would first ask the doctor if I’m able to… I’ll trust him and go wherever he says.”

### Policy Recommendations and Strategies to Improve COVID-19 Vaccine Equity, Trust, and Accessibility

Participants offered and endorsed recommendations or strategies for improved vaccine confidence and accessibility (Table 3). Recommendations include using community engagement, improving empathetic bidirectional deliberation, ensuring timely access to critical information, promoting altruistic and culturally congruent messaging, increasing data transparency, translation, and data collection for diverse populations, and increasing accessibility through navigational and logistical vaccination support.

**Table 3.** Policy Recommendations and Strategies to Improve COVID-19 Vaccine Equity, Trust, Confidence, and Accessibility Endorsed by Racial and Ethnic Minority Participants Using the Vaccine Hesitancy Matrix Categories

Category	Policy recommendations	Strategies
Contextual Influences	Invest in community-based engagement from trusted partners and entities	Empower, employ, and invest in developing vaccine advocates within communities. Known, respected, and trusted community leaders or entities may include community clinics or primary care practitioners, community health workers, faith-based organizations, cultural leaders, schools, and more. Promote microengagement in multigenerational families, tell-one-teach-one campaigns, neighborhoods, or networks. Encourage vaccine deliberation with trusted medical professionals. Provide participatory discussions or safe spaces to provide information and for community members to ask questions or share concerns (eg, community workshops, town halls, and hotlines). Varied pathways for trusted communications should be enacted (internet-alternative methods, mailers, and telephone).
	Provide timely and accessible information from credible sources	Provide accurate updates to combat misinformation, rumors, and myths, especially as COVID-19 news rapidly evolves through social media, news, and current events in local communities. Continuously deliver information through multiple sources (television, radio, social media, and local community spaces) and offer bidirectional venues for communication. Provide information in a community-friendly and accessible format, including infographics, simplified and appropriate language translations and dialects, and culturally relevant information. Communication should be transparent, emphasizing what is known and unknown and validating the importance of asking questions and addressing concerns.
	Reduce structural barriers in vaccine access	Increase accessibility with navigational and logistical support, including translation services, internet-alternative outreach, and transportation. Increase local availability in vaccine allocation, including mobile vaccination clinics, home visits, and other trusted community-based sites (schools, churches, and trusted community organizations). Build on and invest in trusted community networks and organizations that may facilitate communication, outreach, and vaccination logistics for vulnerable individuals.
Individual and group influences	Promote sensitivity and empathy; validate and listen to concerns leading to hesitancy or deliberation	Medical and public health professionals should display heightened awareness, compassion, and understanding for populations with vaccine indecision. Professionals should acknowledge current and historical events, racism, or mistreatment have led to medical and governmental mistrust. Acknowledge the complicity of the medical profession in systemic racism. Communication should not trivialize the pandemic or place blame on communities hesitant or deliberating when delivering information. Sources should portray sensitivity toward those who have faced hardships and loss during the pandemic.
	Promote altruistic and culturally congruent concepts in vaccine messaging	Use communal communication, including for the safety of family, friends, communities and loved ones, and tailored to motives relevant to cultural beliefs and practices. Additional motivators may include reducing anxiety, returning to school, potential for improved employment opportunities or workplace safety, and resuming particular cultural and social norms.
Vaccine- or vaccination-specific issues	Increase the robustness of collection and the transparency and translation of vaccine data for subpopulations	Show research outcomes directly relating to individuals or communities facing particular vulnerabilities who may question vaccine acceptability. Provide vaccine clinical trial outcomes data, including participation demographic characteristics, by race and ethnicity, age, chronic disease status, and disability. Acknowledge what data are unavailable. Data should be accessible to populations of all educational backgrounds. Improve vaccination data collection for subpopulations to understand ongoing vaccine disparities, programming gaps, resource needs, and policy implications. Share strategies, successes, and gaps related to equity within vaccine allocation. Include community members in all aspects of the vaccine development process. Translate scientific findings into accessible, easy-to-understand concepts.

### Community-Partnered Products

Because this was a community-partnered study, we developed COVID-19 vaccine information guides based on the most common questions and concerns described in this study and shared them with all participants. Preliminary results and vaccine-related information were shared with participants, community partners, public health officials, and policymakers through community presentations.

## Discussion

This qualitative study identified factors associated with COVID-19 vaccine decision-making across different racial and ethnic low-income communities in LAC. Themes included fear of differential vaccine treatment, mistrust from unethical research or mistreatment in medicine, inadequate vaccine evidence and information, inequitable allocation and accessibility, and inadequate community engagement efforts. To enhance acceptability and accessibility, disseminating critical information from trusted messengers about population-specific vaccine safety data and providing navigational support to address structural barriers were suggested by participants.

Consistent with prior COVID-19 vaccine acceptability qualitative research, we found information gaps, concerns about the vaccine’s rapid development, and an absence of scientific evidence translated for diverse communities.^11,42,43,44,45^ Culturally centered care and practitioner recommendations may help promote vaccine acceptability, trust, and combat misinformation.^6,46,47^ However, a previous study^47^ found that racial and ethnic minority populations are less likely to receive practitioner recommendations for vaccinations. More recent evidence^48^ suggests inadequate in-language education on COVID-19 information and prevention. Comprehensive messaging of crucial information, particularly about vaccine safety^49^ and efficacy, is needed to make informed decisions and requires critical investment in trusted messengers, including physician recommendations.^1,14^ In particular, our study expanded informational needs to include outcomes data relevant to minority communities, such as population-based participant data in vaccine clinical trials and, when available, effectiveness outcomes by age, race and ethnicity, or chronic disease. Addressing these needs will be particularly important for racial and ethnic minority groups who already face health disparities and fear COVID-19 vaccine adverse effects.

Participants voiced concerns about systemic inequality across the vaccine development and distribution continuum, from clinical trial participation to allocation to mistreatment during vaccination. A previous study^50^ found historical discrimination influences mistrust of health care, including vaccine hesitancy. Furthermore, influences from systematic and institutional mistrust, including widespread uncertainty stemming from the pandemic and recent social justice movements, can create additional obstacles for vaccine uptake.^42,51,52,53,54,55,56,57^ A lack of equity, transparency, or outreach to repair breaches in trust may further erode confidence in the COVID-19 vaccine and health care more broadly.

Because racial and ethnic minority communities are disproportionately affected by social factors that worsen COVID-19–related disparities,^9,43,58,59^ it is critical to identify the societal conditions and harms that lead to these unique vaccination barriers.^8,9,11^ Participants in our study highlighted the difference between equity and equality in vaccine distribution, often referencing concerns about personal, family, or community members who might have difficulty accessing the vaccine without additional attention to availability and cost of transportation, technology assistance, access to medical recommendations, time off from work, and more. Unfortunately, many of these misgivings were realized in the early phases of the vaccine distribution, which has been marred by allocation and uptake disparities, resulting in the underrepresentation of racial and ethnic minority individuals, low-income communities, and essential workers.^60^

The recommendations from study participants to improve vaccine acceptability and accessibility in their communities can support building long-term trust in health care, the scientific process, and public-facing governmental and institutional systems. A multipronged approach to building vaccine acceptability in disenfranchised communities will require local, community-centered actions and activities that develop an understanding of hesitancy, build trust, promote deliberation, and translate findings to national initiatives and policy improvements. National funding initiatives, such as the National Institutes of Health Community Engagement Alliance and Rapid Acceleration of Diagnostics, have promoted community engagement to reduce COVID-19 disparities by developing and disseminating prevention strategies, including clinical trial participation and vaccine or therapeutic uptake.^61,62^

### Limitations

This study has limitations. Its primary intent was to describe common needs and potential areas for intervention for COVID-19 vaccine rollout in high-risk communities in LAC; thus, we did not compare or quantify specific differences across racial and ethnic groups or by age. Future comparative research should examine differences among communities to elucidate how best to tailor interventions. Our findings may not be generalizable to other high-risk groups or geographic areas. Virtual participation requirements may have led to a selection bias against those with limited telephone or internet access, although we offered tablets and Wi-Fi access. This study started before vaccines received US Food and Drug Administration emergency use authorization and ended before vaccines became widely available.^63^ During the study period, COVID-19 vaccine knowledge and awareness shifted. However, findings provide real-time insight into community knowledge, concerns, questions, hopes, and barriers related to the COVID-19 vaccines, potentially future vaccines, and therapies.

## Conclusions 

Understanding factors that influence the multifaceted decision-making process for vaccine uptake in vulnerable communities is critical for narrowing the racial and ethnic as well as socioeconomic disparities observed during the COVID-19 pandemic. Although this study highlights the continual unanswered need in the medical literature to determine the spectrum of mistrust and the interplay with health care decision-making,^64,65^ members of these communities described wide-ranging structural barriers, disparate reasons for historical and contemporary trauma, and insufficient engagement for COVID-19 vaccine accessibility. Participants in this study identified interrelated factors that contribute to COVID-19 vaccine decision-making while emphasizing the importance of equitable access and the parallel need for community engagement in building long-term trust in health care, the scientific process, and public-facing governmental and institutional systems among historically and currently marginalized populations.

## Notes

Supplement. **eTable 1.** Focus Group Participant Demographics **eTable 2.** Focus Group Participant Survey, Reasons for and Against Obtaining Vaccination
