# Omission in the Funding and Support Section

**DOI:** 10.1001/jamanetworkopen.2025.16428

**Published:** 2025-05-19

In the Original Investigation titled “COVID-19 Vaccine Decision-making Factors in Racial and Ethnic Minority Communities in Los Angeles, California,”^1^ which was published on September 30, 2021, a funding source was omitted. The Funding/Support should be as follows: “This research was funded in part by the National Institutes of Health (NIH) agreement OT2HL158287, grant UL1TR001881 from the National Center for Advancing Translational Science, and grant OCRC 20-51 from UCLA.” In addition, a disclaimer was added to indicate that the views and conclusions contained in the article are those of the authors and should not be interpreted as representing the official policies, either expressed or implied, of the NIH. The article has been corrected.
