# Supplementary material for: COVID-19 Vaccine Decision-making Factors in Racial and Ethnic Minority Communities in Los Angeles, California
Source: JAMA Netw Open. 2021 Sep 30;4(9):e2127582. doi: 10.1001/jamanetworkopen.2021.27582 (PMC8485164; doi:10.1001/jamanetworkopen.2021.27582)
Supplement: Supplement. — eTable 1. Focus Group Participant Demographics eTable 2. Focus Group Participant Survey, Reasons for and Against Obtaining Vaccination [file jamanetwopen-e2127582-s001.pdf]

Supplementary Online Content

Carson SL, Casillas A, Castellon-Lopez Y, et al. COVID-19 vaccine decision-making factors in racial and ethnic minority communities in Los Angeles, California. *JAMA Netw Open*. 2021;4(9):e2127582. doi:10.1001/jamanetworkopen.2021.27582

eTable 1. Focus Group Participant Demographics

eTable 2. Focus Group Participant Survey, Reasons for and Against Obtaining Vaccination

This supplementary material has been provided by the authors to give readers additional information about their work.

© 2021 Carson SL et al. *JAMA Network Open.*

**eTable 1.** Focus Group Participant Demographics						
	**All Groups N = 70**	**Black/ African American* N = 17**	**American Indian* N = 17**	**Filipino/ Filipina** N = 11**	**Latino/ Latina* N = 15**	**Pacific Islander*** N = 10**
	No. (%)	No. (%)	No. (%)	No. (%)	No. (%)	No. (%)
**Age**						
20-34	17 (24.29)	6 (35.29)	1 (5.88)	6 (54.55)	2 (13.33)	2 (20.00)
35-49	21 (30.00)	2 (11.76)	6 (35.29)	5 (45.45)	6 (40.00)	2 (20.00)
50-64	22 (31.43)	4 (23.53)	9 (52.94)	0 (0.00)	5 (33.33)	4 (40.00)
65+	10 (14.29)	5 (29.41)	1 (5.88)	0 (0.00)	2 (13.33)	2 (20.00)
**Gender**						
Female	50 (71.43)	15 (88.24)	12 (70.59)	5 (45.45)	10 (66.67)	7 (70.00)
Male	19 (27.14)	2 (11.76)	5 (29.41)	6 (54.55)	5 (33.33)	2 (20.00)
Other	1 (1.43)	0 (0.00)	0 (0.00)	0 (0.00)	0 (0.00)	1 (10.00)
**Education**						
Some High School	5 (7.14)	0 (0.00)	0 (0.00)	0 (0.00)	5 (33.33)	0 (0.00)
High School graduate/GED	5 (7.14)	0 (0.00)	2 (11.76)	1 (9.09)	2 (13.33)	0 (0.00)
Associate's/technical degree	17 (24.29)	3 (17.65)	5 (29.41)	3 (27.27)	5 (33.33)	1 (10.00)
Bachelor's degree	19 (27.14)	6 (35.29)	6 (35.29)	3 (27.27)	2 (13.33)	2 (20.00)
Graduate degree	22 (31.43)	8 (47.06)	4 (23.53)	4 (36.36)	1 (6.67)	5 (50.00)
Prefer not to answer	2 (2.86)	0 (0.00)	0 (0.00)	0 (0.0)	0 (0.00)	2 (20.00)
**Number of People in Household, Mean (SD)**	3.04 (2.17)	2 (1.06)	2.06 (1.20)	3.73 (1.90)	3.93 (1.59)	3.73 (1.90)
**Employment Status**						
Full time	31 (44.29)	6 (35.29)	12 (70.59)	4 (36.36)	2 (13.33)	7 (70.0)
Part-time	18 (25.71)	4 (23.53)	3 (17.65)	6 (54.55)	5 (33.33)	0 (0.00)
Unemployed	13 (10.00)	2 (11.76)	2 (11.76)	1 (9.09)	7 (46.67)	1 (10.00)
Retired	8 (11.43)	5 (29.41)	0 (0.0)	0 (0.0)	1 (6.67)	2 (20.0)
**Essential Worker**	34 (48.57)	8 (47.06)	9 (52.94)	3 (27.27)	8 (53.33)	7 (70.00)
**Resides within a low-income zip code** (Median household income <$40K, per U.S. Census 2010)	39 (55.71)	11 (64.71)	9 (52.94)	4 (36.36)	11 (73.33)	4 (40.0)
**Very Important or Important for all people in community to receive the COVID-19 vaccine**	54 (77.14)	7 (41.18)	13 (76.47)	11 (100.00)	15 (100.00)	8 (80.00)
**Very Likely or Moderately Likely to get an approved COVID-19 vaccine when available**	37 (52.86)	4 (23.53)	8 (47.06)	9 (81.81)	6 (40.00)	10 (100.00)
**COVID History**						
I have previously tested positive for COVID-19, a belief I had COVID-19, or had COVID-19-like symptoms	10 (14.29)	1 (5.88)	2 (11.76)	2 (18.18)	3 (33.33)	2 (20.00)
No, no symptoms of COVID-19	57 (81.43)	16 (94.12)	13 (76.47)	9 (81.82)	12 (80.0)	7 (70.00)
Unsure	3 (4.29)	1 (5.88)	2 (11.76)	0 (0.00)	0 (0.00)	0 (0.00)

Abbreviation: GED: General Education Development.
*3 Focus Groups: 1 >50 years of age, 1 <50 years of age, 1 mixed age
**2 Focus Groups: Mixed age
***2 Focus Groups: 1 >50 years of age, 1 <50 years of age

© 2021 Carson SL et al. *JAMA Network Open.*

eTable 2. Focus Group Participant Survey, Reasons for and Against Obtaining Vaccination

	All Groups N = 70	Black/ African American* N = 17	American Indian* N = 17	Filipino/ Filipina** N = 11	Latino/ Latina* N = 15	Pacific Islander*** N = 10
	No. (%)	No. (%)	No. (%)	No. (%)	No. (%)	No. (%)
**Top reasons obtaining a COVID-19 vaccine** (check all that apply)						
I want to keep my family safe	52 (74.29)	8 (47.06)	10 (58.82)	11 (100.00)	14 (93.33)	9 (90.00)
I want to keep my community safe	44 (62.86)	7 (41.18)	10 (58.82)	7 (63.64)	12 (80.00)	8 (80.00)
I want to keep myself safe	43 (61.43)	7 (41.18)	9 (52.92)	7 (63.64)	12 (80.00)	8 (80.00)
I want to feel safe around other people	39 (55.71)	6 (35.29)	8 (47.06)	7 (63.64)	10 (66.67)	8 (80.00)
I believe life won't go back to normal until most people get a COVID-19 vaccine	38 (54.29)	6 (35.29)	8 (47.06)	7 (63.64)	11 (73.33)	6 (60.00)
I don't want to get really sick from COVID-19	36 (51.43)	6 (35.29)	6 (35.29)	8 (72.73)	9 (60.00)	7 (70.00)
I have a chronic health problem, like asthma or diabetes	14 (20.00)	1 (5.88)	5 (29.41)	3 (27.27)	2 (13.33)	3 (30.00)
My doctor told me to get a COVID-19 vaccine	6 (8.75)	1 (5.88)	1 (5.88)	1 (9.09)	3 (20.00)	0 (0.00)
Other	8 (11.43)	5 (29.41)	2 (11.75)	0 (0.00)	0 (0.00)	1 (10.00)
N/A	2 (2.86)	2 (11.76)	0 (0.00)	0 (0.00)	0 (0.00)	0 (0.00)
**Top reasons for not obtaining a COVID-19 vaccine** (check all that apply)						
I'm concerned about side effects from the vaccine	43 (61.43)	12 (70.59)	10 (58.82)	7 (63.64)	10 (66.67)	4 (40.00)
I don't know enough about how well a COVID-19 vaccine works	41 (58.57)	15 (88.24)	9 (52.92)	6 (54.55)	6 (40.00)	5 (50.00)
I don't trust that the vaccine will be safe	31 (44.29)	9 (52.94)	10 (58.82)	4 (36.36)	7 (46.67)	1 (10.00)
Other	14 (20.00)	5 (29.41)	3 (17.65)	0 (0.00)	4 (26.67)	2 (20.00)
I don't want to pay for it	10 (14.29)	0 (0.00)	0 (0.00)	5 (45.45)	4 (26.67)	1 (10.00)
I don't think vaccines work very well	5 (7.14)	2 (7.14)	0 (0.00)	2 (18.18)	1 (6.67)	0 (0.00)
I'm not concerned about getting really sick from COVID-19	5 (7.14)	3 (17.65)	0 (0.00)	1 (9.09)	1 (6.67)	0 (0.00)
I don't believe the COVID-19 pandemic is as bad as some people say it is	4 (5.71)	2 (11.76)	0 (0.00)	0 (0.00)	2 (13.33)	0 (0.00)
I'm allergic to vaccines.	3 (4.29)	0 (0.00)	1 (5.88)	0 (0.00)	0 (0.00)	2 (20.00)
I don't like needles	3 (4.29)	0 (0.00)	0 (0.00)	1 (9.09)	2 (13.33)	0 (0.00)
N/A	4 (5.71)	0 (0.00)	0 (0.00)	0 (0.00)	0 (0.00)	0 (0.00)

*3 Focus Groups: 1 >50 years of age, 1 <50 years of age, 1 mixed age

**2 Focus Groups: Mixed age

***2 Focus Groups: 1 >50 years of age, 1 <50 years of age

© 2021 Carson SL et al. *JAMA Network Open.*
